# Ahmed glaucoma valve implantation in a case of volatile intraocular pressure and preoperative hypotony

**DOI:** 10.1016/j.ajoc.2025.102285

**Published:** 2025-02-18

**Authors:** Blake Katsev, Gio Campagna, James C. Liu

**Affiliations:** Department of Ophthalmology and Visual Sciences, Washington University School of Medicine, St. Louis, MO, USA

**Keywords:** Ahmed glaucoma valve, Glaucoma drainage implants, Glaucoma tube shunt

## Abstract

**Purpose:**

To describe a case of Ahmed glaucoma valve (AGV) implantation in a patient with clinical hypotony. In this instance, the AGV helped to stabilize labile intraocular pressures (IOP) by introducing a new outflow pathway that could buffer against variable aqueous inflow.

**Observation:**

Our patient presented with severe mixed mechanism glaucoma in his only seeing eye and had a history of failed prior angle surgeries, filtration surgery, and cyclodestructive procedures. After a recent diode, the patient's IOP was difficult to control and ranged from 2 mmHg on a single topical medication to 28 mmHg with no medications. Preoperatively, the patient had visually significant hypotony (corneal and macular folds) while on one topical agent. An AGV was implanted with subsequent stabilization of IOP and vision after postoperative week 6 without the need for IOP lowering agents. The IOP and vision have maintained stability after greater than 1 year of follow-up.

**Conclusion and Importance:**

When the outflow pathways are compromised, any fluctuation in the inflow has a much more dramatic effect on the IOP. In such instances, IOP lowering agents have a very narrow therapeutic window and lead to volatility in IOP. This case demonstrates the potential for tube shunt surgery to reduce the lability of IOP in a patient with volatile IOP control.

## 1 Introduction

Glaucoma is marked by optic nerve damage in the setting of elevated intraocular pressure (IOP). Severe disease has been associated with more volatile and labile pressures due in part to conventional outflow dysfunction, which becomes easily overwhelmed with increases in aqueous fluid production.^1^ Common strategies to reduce IOP include aqueous suppressants or cyclodestructive procedures that decrease aqueous production. However, IOP fluctuations can persist because the outflow pathway may remain noncompliant and dysfunctional.

An alternative procedure to reduce pressure is the introduction of a secondary outflow pathway, which can accommodate larger IOP variations. Glaucoma tube shunts are typically not part of the treatment paradigm in the management of hypotony because their principal purpose is to reduce IOP.^2^^,^^3^ However, in patients whose pressures are particularly labile, i.e. above target without topical therapies but hypotonous with them, the placement of a glaucoma tube shunt may provide IOP stability. We theorized that in an eye with both poor aqueous production and aqueous outflow, a secondary outflow pathway with a valved tube shunt may stabilize IOP volatility.

## 2 Case report

A 71-year-old African American male with a past medical history of diabetes, hypertension, and kidney dysfunction presented for routine clinical care. His right eye had light perception visual acuity (VA) due to a central retinal vein occlusion and subsequent neovascular glaucoma. His better seeing left eye had severe mixed-mechanism glaucoma caused by a history of trauma, uveitis, and steroid-induced IOP elevations. Prior to presentation, he received a trabeculectomy that scarred and failed shortly afterwards. Subsequently, he was managed with topical therapies.

**Fig. 1:**
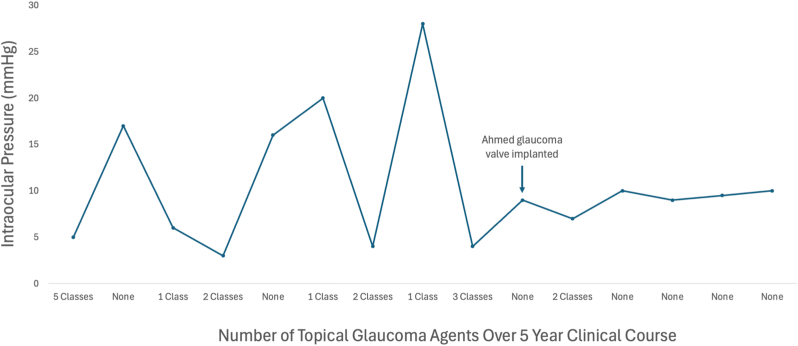
IOP trend prior to and after surgery with Ahmed glaucoma valve. Associated medication requirements (number of IOP lowering classes) are shown.

**Fig. 2:**
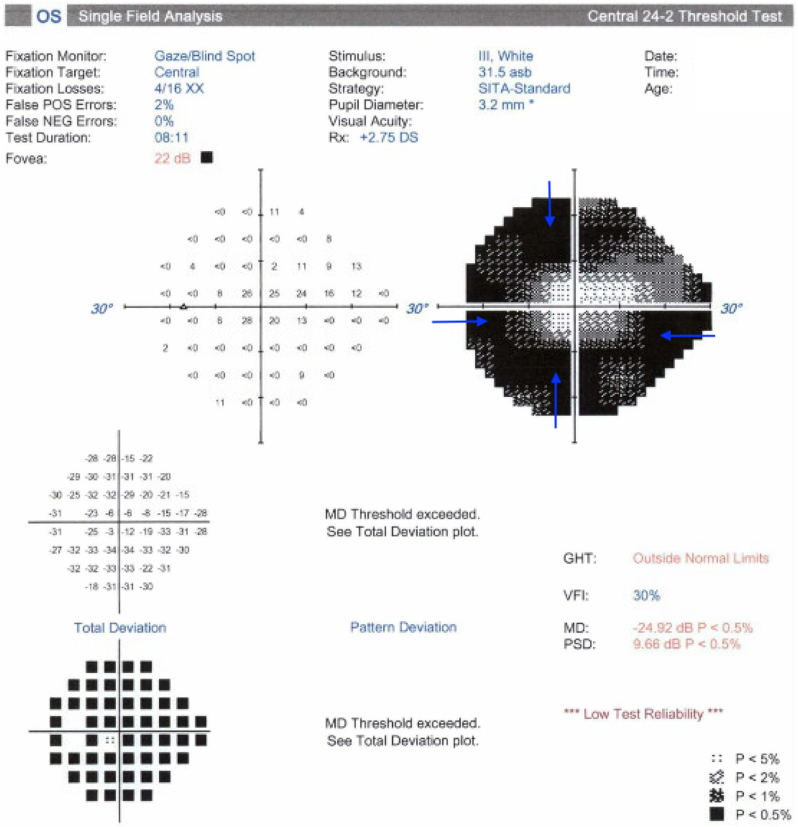

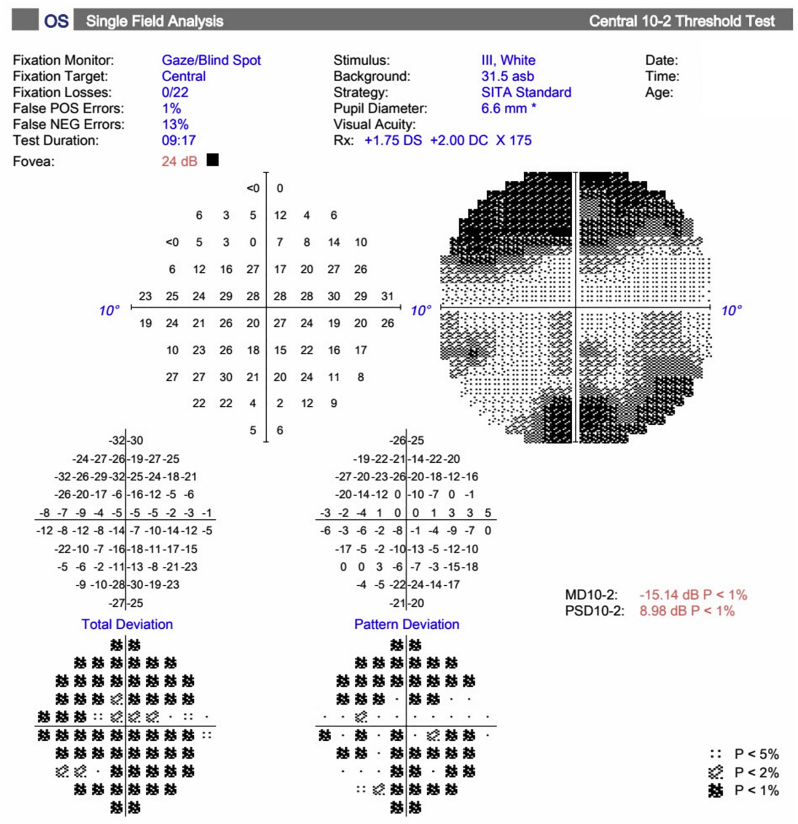

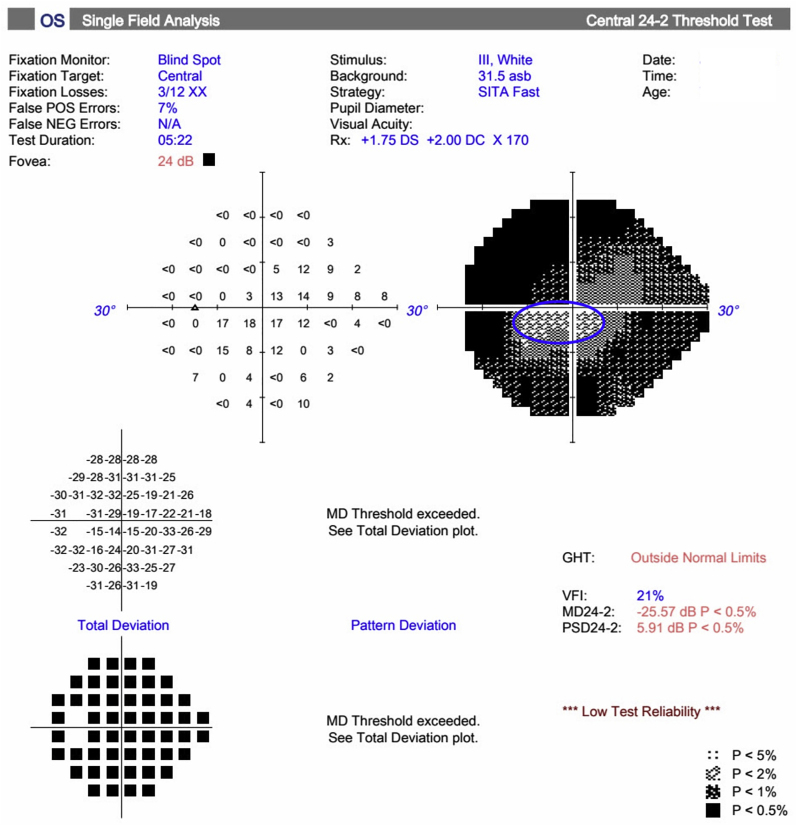
Humphrey Visual Field (HVF) of the left eye demonstrating progression of disease. (a) Baseline HVF 24-2 in 2019 demonstrating significant inferior and superior arcuate scotomata consistent with severe glaucoma, (b) HVF 10-2 in 2021 demonstrating glaucomatous visual field loss near fixation with an inferotemporal central island, and (c) HVF 24-2 in 2023 demonstrating progression of glaucomatous visual field loss with only a small inferotemporal island of central vision remaining.

However, due to rising IOP and progression on visual fields, he underwent diode cyclophotocoagulation. On postoperative month one, the patient was hypotonous with an IOP of 3 mmHg and symptomatic macular folds, reducing his VA from 20/80 to 20/150. Topical therapies were weaned off, which lead to improvement of VA, but IOP became well above goal. Subsequently, multiple combinations of topical therapies were used in an attempt to reach a goal IOP without producing symptoms of hypotony. Over a 4-year period, various combinations of drops and dosing frequencies were attempted, including netarsudil 0.02 % (nightly), netarsudil-latanoprost 0.02–0.005 % (nightly), brimonidine 0.2 % (twice daily), dorzolamide-timolol 22.3–6.8 mg/mL (once daily; twice daily), travoprost 0.004 % (nightly), latanoprost 0.005 % (nightly), and timolol 0.5 % (daily; twice daily). However, IOP became extremely sensitive to topical therapies and produced a volatile IOP trend (Fig. 1). During episodes of low pressure, the patient would notice vision blur and metamorphopsias. The patient was largely asymptomatic when the pressure was elevated, but eventually developed concerning signs of progression of disease (Fig. 2a–c). Although even a single topical agent would be sufficient to lower the pressure below goal IOP, the therapeutic window for this patient was narrow. Therefore, we were unable to keep him in a range that prevented visual symptoms from hypotony while also delaying progression of disease.

**Fig. 3:**
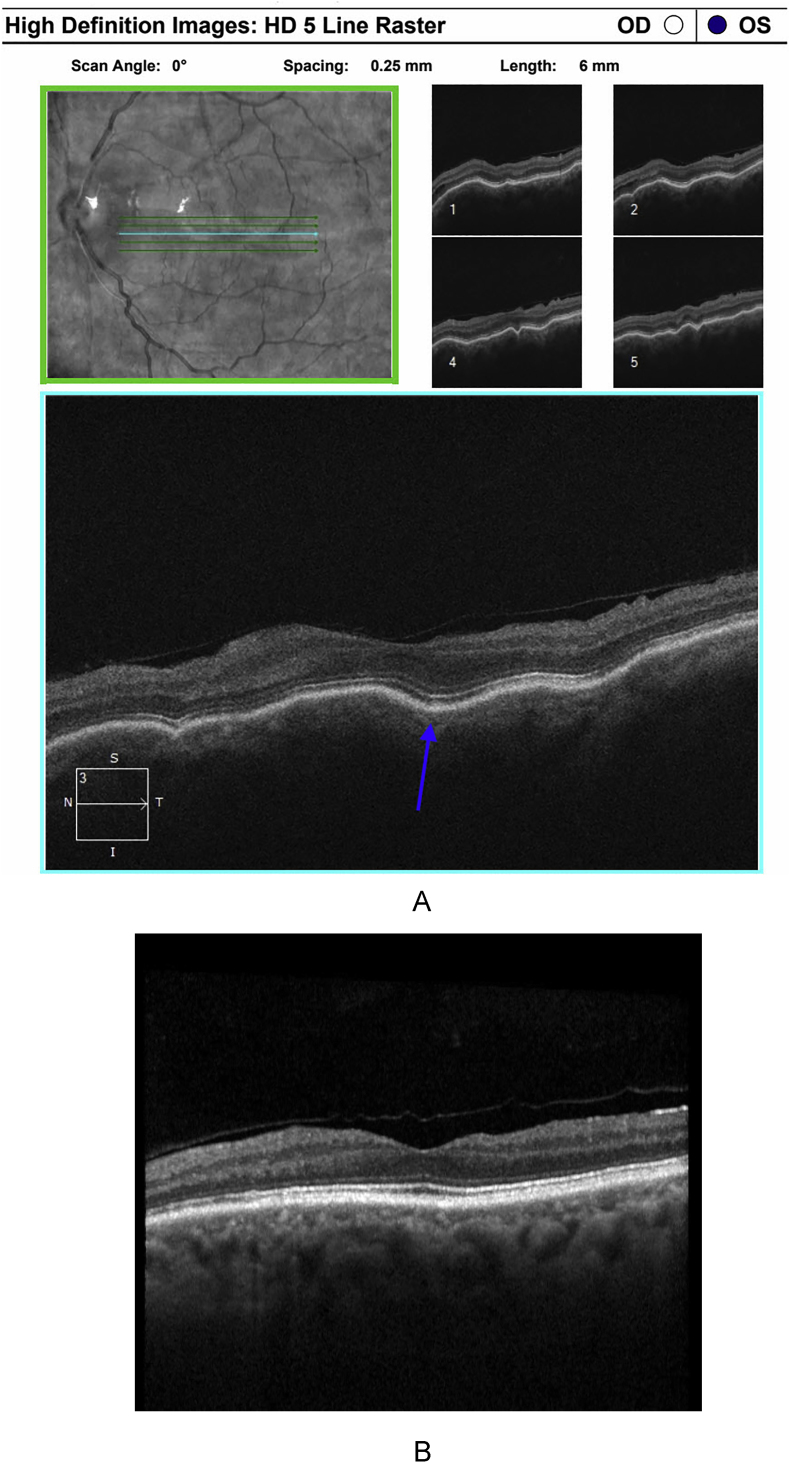
(a) Optical coherence tomography (OCT) of the left eye in 2020 demonstrating visually significant macular folds due to hypotony (solid arrow). (b) 2024 OCT demonstrating the resolution of choroidal folds after valve placement and subsequent IOP stabilization.

Consequently, we decided to place an Ahmed glaucoma valve (AGV) theorizing that the tube shunt would stabilize the labile IOP by creating a compliant outflow system that could buffer against any variable inflow. The operation went uneventfully. Intraoperatively, the tube lumen was ligated with a Vicryl suture with the goal of reducing early postoperative hypotony and to blunt capsule formation. On postoperative day one, his IOP was 9 mmHg, but at postoperative week one, his IOP had increased to 27 mmHg. Due to this increase in IOP, a combination aqueous suppressant was added. Around postoperative week six, his IOP was 7 mmHg, the ligation suture had dissolved, and the aqueous suppressants were all stopped. Since then, multiple follow-up visits have demonstrated IOPs ranging between 8 and 10 mmHg, with resolution of corneal and macular folds on exam and optical coherence tomography (Fig. 3a–b). The patient has maintained stable vision and IOP without need for IOP lowering agents, with the most recent follow-up exam conducted 15 months postoperatively.

## 3 Discussion

Glaucoma and IOP elevation occur when there is a mismatch between aqueous production and aqueous outflow. In the normal eye, a functioning outflow pathway can accommodate for some elevations in aqueous production due to a more compliant drainage system. This allows the IOP to remain in a sufficiently narrow range which prevents damage to the optic nerve. However, in the glaucomatous eye, the outflow pathway is damaged and loses the capacity for homeostasis in IOP regulation.^1^ This damaged regulatory response and the decreased flow rates seen in glaucomatous eyes create more volatile IOP. This is exemplified by studies demonstrating significantly higher diurnal variations of IOP in glaucomatous eyes.^4^, ^5^, ^6^

To lower IOP, one can either decrease inflow or increase outflow. Aqueous suppressants or cyclodestructive procedures can be used to decrease aqueous production and inflow. To increase outflow, bypassing the conventional outflow system with a surgical procedure (trabeculectomy or tube shunt surgery) is often preferred.^7^

Prior to this patient's AGV implantation, the patient's glaucoma was treated medically with aqueous suppressants. However, given the defective outflow pathway, the medical therapies had an extremely narrow therapeutic window for this patient which frequently resulted in symptomatic hypotony while on treatment and elevated IOP while off. The subsequent placement of the AGV created a compliant outflow pathway that buffered against variable inflow and ultimately stabilized the labile IOP despite the patient's history of hypotony.

## 4 Conclusion

This case highlights the need for close surveillance when treating advanced glaucoma by decreasing aqueous production as the sole method. While cyclodestructive procedures are common tools used in the treatment of glaucoma due to their effectiveness in reducing IOP and technical ease of the procedures themselves, utilizing these procedures in patients that have dysfunctional outflow pathways can lead to unpredictable changes in IOP.^8^, ^9^, ^10^ The drainage system can still become overwhelmed and cannot accommodate variable aqueous humor production, which in turn leads to more volatile IOP. In such settings, focusing on bypassing the dysfunctional outflow pathways (i.e., AGV implantation) may be a more efficacious strategy.

## CRediT authorship contribution statement

**Blake Katsev:** Writing – original draft, Visualization, Software, Resources, Formal analysis, Data curation. **Gio Campagna:** Writing – review & editing, Supervision, Methodology, Conceptualization. **James C. Liu:** Writing – review & editing, Supervision, Conceptualization.

## Patient consent

Written consent to publish this case has not been obtained. This report does not contain any personal identifying information.

## Authorship

All authors attest that they meet the current ICMJE criteria for Authorship.

## Funding

There were no funds allocated to the realization of this clinical case.

## Declaration of competing interest

The authors declare that they have no known competing financial interests or personal relationships that could have appeared to influence the work reported in this paper.
